# Hepatopancreas-Intestinal Health in Grass Carp (*Ctenopharyngodon idella*) Fed with Hydrolyzable Tannin or Rapeseed Meal

**DOI:** 10.1155/2022/6746201

**Published:** 2022-12-15

**Authors:** Jingting Yao, Ying Hang, Xueming Hua, Ningyu Li, Xiang Li

**Affiliations:** ^1^Centre for Research on Environmental Ecology and Fish Nutrition (CREEFN) of the Ministry of Agriculture and Rural Affairs, Shanghai Ocean University, Shanghai, China; ^2^Key Laboratory of Freshwater Aquatic Genetic Resources, Ministry of Agriculture and Rural Affairs, Shanghai, China; ^3^National Demonstration Center for Experimental Fisheries Science Education, Shanghai Ocean University, Shanghai, China

## Abstract

This study evaluated the effect of dietary rapeseed meal (RM) and hydrolyzable tannin on grass carp (*Ctenopharyngodon idella*) and determined the possible role of tannin on health when RM was added to the diet. Eight diets were formulated. Four were semipurified-diets with 0, 0.75, 1.25, and 1.75% hydrolyzable tannin (T0, T1, T2, and T3), and the other four were practical diets containing 0, 30, 50, and 70% RM (R0, R30, R50, and R70), which had similar tannin contents as semipurified-diets. After the 56 d feeding trial, the antioxidative enzymes and relative biochemical indexes showed a similar tendency in practical and semipurified groups. In hepatopancreas, superoxide dismutase (SOD) and catalase (CAT) activities increased with RM and tannin levels, respectively, while glutathione (GSH) content and glutathione peroxidase (GPx) activity increased. Malondialdehyde (MDA) content increased and decreased in T3 and R70, respectively. In the intestine, MDA content and SOD activity increased with RM and tannin levels, while GSH content and GPx activity decreased. The expression levels of interleukin 8 (*IL-8*) and interleukin 10 (*IL-10*) were upregulated with RM and tannin levels, and the Kelch-like ECH-associated protein 1 (*Keap1*) expression was upregulated in T3, whereas it was downregulated in R50. This study demonstrated that 50% of RM and 0.75% of tannin induced oxidative stress, injured hepatic antioxidant ability, and resulted in intestinal inflammation in grass carp. Therefore, the tannin in rapeseed meal cannot be neglected in aquatic feeding.

## 1. Introduction

With the proportion of fishmeal in aquafeeds declining significantly, it is extremely important to seek alternatives to fishmeal. Rapeseed meal (RM) is an essential source of aquatic protein with a relatively well-balanced amino acid profile and protein concentration (320-450 g/kg dry matter) [1]. RM, which has been evaluated as a substitute for fishmeal in aquafeed application research over the past decade, is considered a valuable protein source for fishmeal-free feeds [2–6]. However, the various antinutritional factors (ANFs) contained in it affect the inclusion of rapeseed meal as a fishmeal substitute in aquafeeds. ANFs (including glucosinolates, phytic acid, and tannin) are responsible for these since it can impede nutrition metabolism, harm organ health, and alter growth performance [7, 8].

As essential antinutritional factors in RM, tannins bind to dietary components, such as proteins, vitamins, and minerals, to reduce digestibility. Hydrolyzable tannin is destroyed in biological systems, creating smaller molecules that enter the bloodstream and cause toxicity in organs (e.g., liver and kidney) [9]. In several animal studies, different effects of hydrolyzable tannin were reported. In previous studies, 5.0 g/kg of dietary hydrolyzable tannin improved the intestinal health of chicken by lowering intestinal skatole formation and regulating necrotic enteritis, while no favorable effect on intestinal morphology of male pigs was observed when 3% of the dietary hydrolyzable tannin-rich extract was supplemented [10, 11]. Most studies on tannin have been limited to mammals, and fewer studies have been conducted on aquatic animals. A study in our lab found that 1.25% of dietary hydrolyzed tannins caused damage to the liver health of puffer fish (*Takifugu obscurus*) [12]. Other results have shown that the presence of tannins in the diet of grass carp can cause damage to its hepatopancreas [13]. In addition, in carp, tannins were found to inhibit antioxidant enzymes and damage polyribosomes in the liver [14]. However, current studies on the effects of tannins on fish health use artificial tannins additives, and their effects on fish may differ from those of naturally occurring tannins.

Liver, which plays an important role in the detoxification, accumulation, and biotransformation of xenobiotics, is easy to be adversely influenced once the health of animals is injured. In Nile tilapia, 15-75% of dietary RM was used. With the increase of RM, the liver structure was damaged, appearing the hepatocytes with empty vacuoles degeneration and nuclear migration [15]. The hepatobiliary tract, which participates in various metabolic processes, such as bile acid secretion and circulation, intestinal motility, intestinal microbiota composition, and nutrient absorption, links the intestine and the liver [16]. Oxidative stress and inflammation are important indicators of hepatic and intestinal injury. The intestine is the main organ for absorbing nutrition, and it is also a significant immune organ for preserving fish health. The thickness of the intestine in fish is thinner than that in mammals, and its contact with the environment and direct digestion makes the aquatic intestine vulnerable, making the integrity of the digestive tract extremely important [17]. There are injury and inflammation in the intestine, especially in the hindgut, when damage occurs in the digestive tract of fish [18]. In grass carp (*Ctenopharyngodon idella*) studies, it was found that after feeding high rapeseed meal diet, villi height, V/C, and goblet cell values decreased, and the tips of intestinal villi were relatively thin [19].

Interleukin 8 (*IL-8*) and interleukin 10 *(IL-10*) are important interleukins, which play a pivotal part in inflammation. *IL-10* is an anti-inflammatory factor, and *IL-8* is a proinflammatory factor. The transcriptional expression of these interleukins can indicate the condition of inflammation [20]. One of the most important cellular defense mechanisms against oxidative stress is the nuclear factor erythroid 2-related factor 2 (*Nrf2*)-Kelch-like erythroid cell-derived protein with CNC homology- (ECH-) associated protein 1 (*Keap1*) system. *Nrf2* regulates the expression of numerous antioxidant genes, and it is a primary target of *Keap1* [21]. Nutrients and ANFs may have an impact on fish health, but little is known regarding the link between hydrolyzable tannin, rapeseed meal, and fish health.

Grass carp is favored by fish producers. As a native Chinese carp, it is the most productive species of freshwater fish in China. Its total output was 5571 thousand tons in 2020 [22], accounting for nearly 1/5 of the total freshwater fish production in China. As an herbivorous species, it tolerates a higher level of plant materials compared to carnivorous fish. The percentage of dietary rapeseed meal in grass carp diets could be closer to 35% [23, 24]. The tannin content of rapeseed meal may be used to predict the tolerance of the given aquatic animal to other tannin-containing plant feedstuffs and single tannin. This depends to a great extent on the mechanisms of different forms of tannin in the aquatic animals, and it is still unclear for grass carp, although studies on the gradient of hydrolyzable tannins in grass carp's feed have provided some evidence of changed nutrient digestibility and utilization and intestinal microbiota profile [25, 26].

In the present study, relative expression of several cytokines, signaling pathway of a nuclear factor in the intestine, antioxidant enzyme activities, and immune parameters in hepatopancreas, intestine, serum, and head kidney was detected to evaluate the effect of dietary RM and tannin on the health and antioxidative ability in grass carp. Furthermore, the histological change of the hindgut was observed to evaluate intestinal health and clarify the influence of dietary RM and tannin on intestinal stress response in grass carp. By comparing the RM-used practice and tannin-contained semipurified diets, this study determined whether tannin played the main role in fish health when RM was used in the diet of grass carp.

## 2. Materials and Methods

### 2.1. Experimental Animal and Setting Conditions

The experiment was conducted at the Shanghai Ocean University Coastal Aquaculture Station, China. Huzhou Nanxun Honghao Fisheries provided grass carp with an average weight of 8.18 ± 0.81 g. Fish were randomly placed into 32 400 L-cages (0.7 m^∗^0.8 m^∗^0.8 m) fixed in indoor tanks (four cages per tank). Fish were randomly divided into eight groups with four replicates (20 fish per group). For a one-week acclimation phase, the fish were fed commercial meals. Throughout the trial, environmental and water quality indicators were evaluated. The tanks received filtered pond water and continuous aeration to maintain a suitable amount of dissolved oxygen (DO) (>5 mg/L) and ammonium nitrogen (NH_3_-N) (<0.6 mg/L). The water temperature ranged from 24-32°C. Every five days, the water was changed, and filtered pond freshwater replaced 1/3 of it. All protocols complied with national and institutional guidelines for the care and use of experimental animals. The handling and treatment of experimental fish were carried out in line with the procedures established by the Shanghai Ocean University (SHOU) Institutional Animal Care and Use Committee (IACUC), and this work was authorized by the IACUC of SHOU, Shanghai, China.

### 2.2. Diet and Feeding

The feeding trial lasted for eight weeks, and all fish were fed three times a day to apparent satiation (7 : 00, 12 : 00, and 17 : 00). Uneaten diet was collected 30 min after feeding by siphoning, then dried and weighed to adjust feed intake. The feed ration was varied during the study as the animals' appetites altered. Eight isonitrogenous (crude protein of 30.00-31.18%) and isoenergetic diets (gross energy of 16.73-17.63 MJ/kg) were formulated. Four of them were semipurified diets containing 0% (T0), 0.75% (T1), 1.25% (T2), and 1.75% (T3) hydrolyzable tannin, casein, and gelatin as the main protein sources and soybean oil as the main lipid source (Table 1). The other four diets were practical diets with 0% (R0), 30% (R30), 50% (R50), and 70% (R70) RM, containing similar total tannin content to semipurified diets. The major protein sources were fishmeal, soybean meal, and rapeseed meal, with soybean oil as the major lipid source (Table 2).

All ingredients were processed through a 60-mesh sieve. The individual ingredients were weighed according to the formula. Soybean oil and the appropriate amount of water (25%-30%) were added, mixed, and pelleted (1.5 mm diameter) in an experimental feed pelletizer and then dried in a blast drying oven at 40°C for approximately 12 h. The dried rations were broken and sieved to the appropriate pellet size and stored in sealed bags in a cold, dry, well-ventilated place until use.

### 2.3. Sample Collection

All fish in each cage were counted and weighed at the end of the 56 d feeding period. Prior to dissection, seven fish were randomly selected from each cage (a total of 28 fish per group) and sedated with eugenol solution (100 ppm). The surface of the fish was wiped clean. The abdomen was split along the middle. The weights of the hepatopancreas, spleen, and intestine were all measured. The following formulas were used to compute weight gain rate (WGR), feed conversion ratio (FCR), feed rate (FR), survival rate (SR), relative intestine weight, relative hepatopancreas weight, and relative spleen weight.

(1) $$\begin{array}{c} \text{WGR}=\left(\text{final weight}-\text{initial weight}\right)\times \frac{100}{\text{initial weight}},\\ \text{FCR}=\text{amount of feed}\frac{\text{intake}}{\text{body mass gain}},\\ \text{FR}=\text{total feeding}\times \frac{100}{\left[\text{feeding period}\times \text{final weight}+\text{initial weight}/2\right]},\\ \text{SR}\left(\%\right)=\text{final number of fish}\times \frac{100}{\text{initial number of fish}},\\ \text{Relative intestine weight}\left(\%\right)=\text{intestine mass}\times \frac{100}{\text{body mass}},\\ \text{Relative hepatopancreas weight}\left(\%\right)=\text{hepatopancreas mass}\times \frac{100}{\text{body mass}},\\ \text{Relative spleen weight }\left(\%\right)=\text{spleen mass}\times \frac{100}{\text{body mass}}. \end{array}$$

Blood samples were drawn from the caudal vein, centrifuged at 4000 × g for 10 min at 4°C, and the serum was obtained from the supernatant and kept in centrifuge tubes at -20°C. The sampled fish's hepatopancreas and intestine were collected on ice before storing at -20°C.

Another three fish were chosen at random from each cage (12 fish per group) for histology observation. The hindgut was extracted and preserved in Bouin's solution for 24 h before processing and embedding in paraffin.

Three more fish were taken from each cage (12 fish per group), sedated, and slaughtered as described above. Under sterile circumstances, the hindguts of the chosen fish were collected. To determine the expression levels of *Nrf2* messenger RNA (mRNA), *Keap1* mRNA, *IL-8* mRNA, and *IL-10* mRNA, the tissues were frozen in RNAse-free centrifuge tubes and kept at -80°C for RNA extraction.

### 2.4. Biochemical Indexes

The hepatopancreas and intestine samples were homogenized with sterile 0.85% saline solution to yield 10% (W: V) homogenates, which were then centrifuged at 4000 × g for 10 min at 4°C. At 12 h, supernatants were utilized to analyze biochemical indexes.

Specific analytical methodologies and commercially available kits were used to measure the biochemical indexes (Jiancheng Bioengineering Institute, Nanjing, China). In serum, superoxide dismutase (SOD), glutamic-pyruvic transaminase (GPT), glutamic oxalacetic transaminase (GOT), and total antioxidative capacity (T-AOC) were measured; in hepatopancreas, the activities of SOD, T-AOC, catalase (CAT), and glutathione peroxidase (GPx) and the content of glutathione (GSH) and malondialdehyde (MDA) were measured; in intestine, the activities of GPx and SOD and content of GSH and MDA were measured; in head kidney, the activities of lysozyme (LZM) and acid phosphatase (ACP) were measured.

### 2.5. Slice Preparation and Intestinal Index Observation

The hindguts were fixed with Bouin's solution for 24 h, processed and embedded in paraffin blocks according to conventional protocols, cut into 7-mm thick slices [27], inserted and stretched, dried at 40°C for 24 h, and dyed. The height of the intestinal folds was measured using light microscopy on slices.

### 2.6. Isolation of Total RNA

Total RNA was isolated from hindguts samples stored at -80°C using the Trizol Reagent, according to the manufacturer's instructions (Invitrogen, Waltham, MA, USA). A UV spectrophotometer was used to determine the RNA concentration according to absorbance at 260 nm (Thermo Fisher Scientific, Waltham, MA, USA). We assessed the purity by measuring the RNA content and the optical density (OD) at 260 nm (OD260)/OD at 280 nm (OD280) ratio. The total RNA samples typically yielded 100 ng/*μ*L RNA and an OD260/OD280 ratio between 1.8 and 2.0.

### 2.7. Real-Time Quantitative Reverse Transcription *PCR* (qRT-PCR)

*β*-Actin (GenBank accession no.: DQ211096), *Nrf2* (GenBank accession no.: KF733814), *Keap1a* (GenBank accession no.: KF811013), *IL-8* (GenBank accession no.: JN663841), and *IL-10* (GenBank accession no.: HQ388294) of grass carp were chosen as reference genes based on conserved gene complementary DNA (cDNA) sequences. Jiangsu Jin Weizhi developed and synthesized fluorescent quantitative primers (Table 3) using Primer5 software (Jiangsu City, China).

The SuperReal SYBR Green (TianGen, Beijing, China, FP205) of the two-step RT-PCR kit was used to perform real-time fluorescence quantitative PCR on the ABI7500 according to the instructions. The reaction system for qRT-PCR was the same as Yao et al. [28]. The relative mRNA expression levels of genes were calculated via the 2^−△△Ct^ method. The T0 and R0 groups were regarded as the control groups in semipurified-diet and practical-diet groups, respectively.

### 2.8. Data Analysis

Means ± standard deviations (SD) were used to show the data. In SPSS version 17.0 (IBM, Chicago, IL, USA), the data were analyzed using a one-way analysis of variance (ANOVA). When ANOVA revealed differences between groups, Duncan's multiple range test was done to investigate differences in mean values.

## 3. Results

### 3.1. Survival and Growth Performance

The SR, WGR, relative hepatopancreas weight, and relative intestine weight did not change significantly among semipurified diet groups (*P* > 0.05; Table 4). T2 had a greater FCR and FR than T1 and T3 (*P* > 0.05), as well as significantly higher FCR and FR than T0 (*P* < 0.05). The relative spleen weight of T0 and T1 was significantly higher than that of T2 and T3 (*P* < 0.05).

Among practical diet groups, SR, WGR, relative hepatopancreas weight, and relative intestine weight did not change significantly (*P* > 0.05; Table 5). The FCR of the R70 group was significantly (*P* < 0.05) higher than that of other groups, and the FR was also highest in the R70 group (*P* < 0.05). The relative spleen weight of R70 was significantly (*P* < 0.05) higher than that of R30 and R50.

### 3.2. Biochemical and Immune Parameters

In semipurified diet groups, in hepatopancreas, the content of MDA increased significantly (*P* < 0.05, Table 6) and then decreased in the T3. The activity of SOD was significantly (*P* < 0.05) lower in the T0 and T1 than that in T2 and T3. The activity of CAT in T3 was higher than that in T2 and T1 and lowest in T0 (*P* > 0.05). The activity of T-AOC of T1 was higher than that of T0, and the activity of T-AOC T0 was higher than that of T2 and T3 (*P* < 0.05). The activity of GP*x* and the content of GSH showed an inverse relationship with dietary tannin (*P* < 0.05).

In serum, the activity of SOD increased and then decreased in the T3 (*P* < 0.05, Table 6). The activity of T-AOC decreased with tannin level and increased in T3 significantly (*P* < 0.05). The activity of GPT was highest in T2. This activity in T2 was higher than that in T0 and T3 (*P* > 0.05) and lowest in T1 (*P* < 0.05). The GOP activity decreased and then significantly increased in T3 (*P* < 0.05).

In intestine, the MDA content and the SOD activity increased with tannin levels significantly (*P* < 0.05, Table 6), while the GP*x* activity and the GSH content showed an opposite change (*P* < 0.05).

In the head kidney, the activity of lysozyme significantly (*P* < 0.05, Table 6) increased with dietary tannin, while the activity of ACP decreased (*P* < 0.05).

In practical diet groups, in hepatopancreas, the content of MDA in the R50 group was significantly (*P* < 0.05, Table 7) higher than that in other groups, and the activity of SOD in the R70 group was significantly (*P* < 0.05) higher than that in other groups. The activity of CAT of R50 and R70 was higher than that of R0 and R30. The activity of T-AOC of R50 was higher than R30 and lowest in R0 and R70 (*P* < 0.05). The content of GSH showed an inverse relationship with the dietary RM (*P* < 0.05). The activity of GP*x* increased and then decreased in R50 and R70 (*P* < 0.05).

In serum, the activity of SOD in the R70 group was higher than that in other groups (*P* < 0.05, Table 7), while the GPT showed an opposite result. The activity of GOP in the R70 was higher than that in R30 and lowest in R0 (*P* < 0.05), and the activity of T-AOC decreased with dietary RM significantly (*P* < 0.05).

In intestine, the MDA content and the SOD activity increased significantly (*P* > 0.05, Table 7), while the GP*x* activity showed an inverse relationship with dietary RM. The GSH content in R0 was higher than that in R50, and the GSH content in R50 was higher than that in R30 and lowest in R70 (*P* < 0.05).

In the head kidney, the activity of lysozyme in R50 and R70 was significantly (*P* < 0.05, Table 7) higher than that in R0 and R30, and the activity of ACP showed an inverse relationship with dietary RM (*P* < 0.05).

### 3.3. Histomorphology of Hindgut and Reactions of mRNA Gene Expressions in the Hindgut

**Figure 1:**
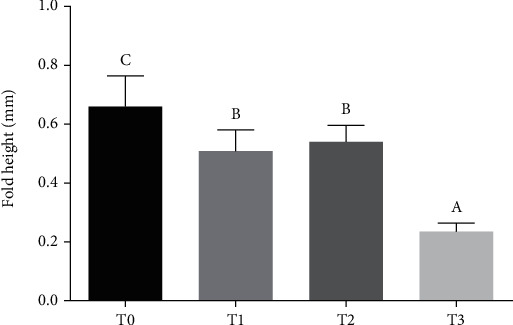
Fold height of hindgut in grass carp fed semipurified diets (mm) (means ± SD, *n* = 10). Note: different lower-case letters indicate significant differences (*P* < 0.05).

In semipurified diet groups, in the hindgut, the height of the intestine folds of the T0 group was significantly higher than that in the T1 and T2 groups (*P* < 0.05, Figure 1) and lowest in the T3 group (*P* < 0.05).

**Figure 2:**
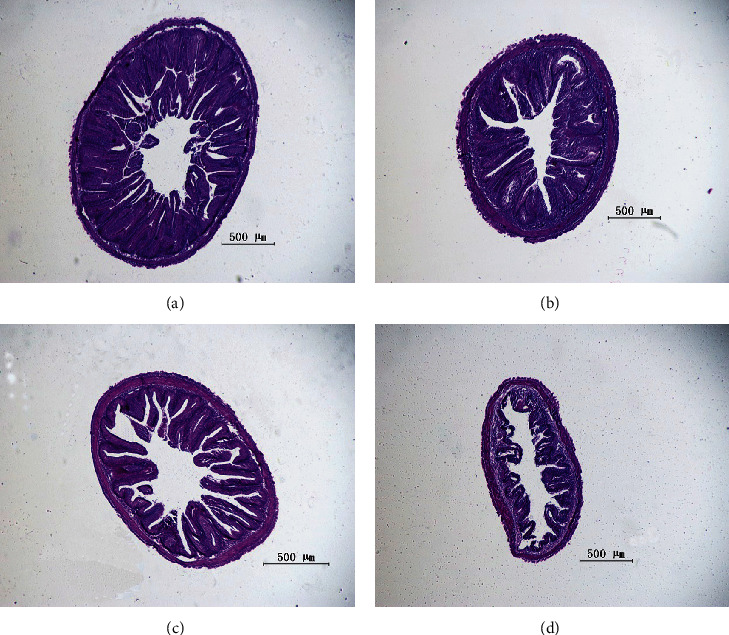
Hindgut morphology of grass carp fed semipurified diets. (a) the hindgut morphology of T0; (b) the hindgut morphology of T1; (c) the hindgut morphology of T2; (d) the hindgut morphology of T3.

As shown in Figure 2(a), the integrated epithelial structure was developed, and tight intestinal villi were observed in the T0 group. In Figure 2(b), the structure of the hindgut was integrated, but several gaps in the mucosal brush border were obtained in the T1 group. In Figure 2(c), the mucosal brush border of the T2 group contained gaps, and the intestinal villi were disrupted, while the intestinal tissue structure was injured in the T3 group (Figure 2(d)), which included atrophy and tattered structure of folds, and the intestinal villi were sparse.

In the hindgut, the relative expression levels of *IL-8* mRNA and *Nrf2* mRNA in the T0 group were significantly lower than those in other groups (*P* < 0.05, Table 8). The relative expression of *IL-10* mRNA upregulated with dietary tannin increased (*P* < 0.05, Table 8). The relative expression of *Keap1* in T2 was significantly higher than T1 (*P* < 0.05). The relative expression of *Keap1* in T1 was higher than that in T3 and lowest in T0 (*P* < 0.05, Table 8).

**Figure 3:**
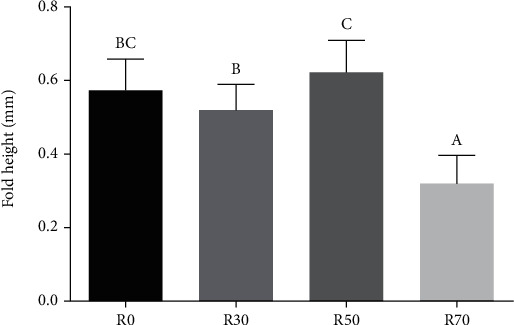
Fold height of hindgut in grass carp fed practical diets (mm) (means ± SD, *n* = 10). Notes: as above.

In practical diet groups, in the hindgut, the height of the intestine folds of the R50 group was higher than that of the R0 group (*P* > 0.05, Figure 3), and the height of the intestine folds of the R0 group was higher than the R30 group (*P* > 0.05) and lowest in R70 group (*P* < 0.05).

**Figure 4:**
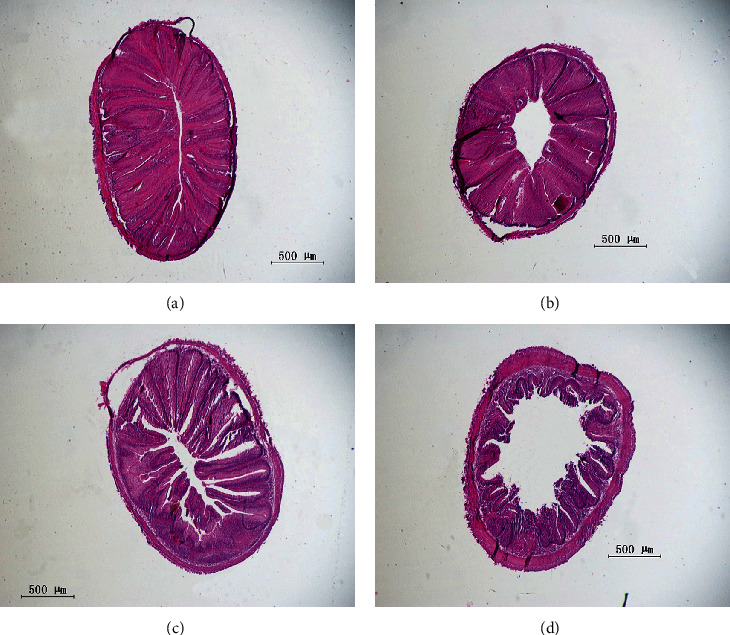
Hindgut morphology of grass carp fed practical diets. (a) the hindgut morphology of R0; (b) the hindgut morphology of R30; (c) the hindgut morphology of R50; (d) the hindgut morphology of R70.

As shown in Figures 4(a) and 4(b), the integrated epithelial structure was developed, and tight intestinal villi were found in the R0 and R30 groups. In Figure 4(c), the mucosal brush border of the R50 contained gaps, and the intestinal villi were disrupted, while in the R70 (Figure 4(d)), the intestinal tissue structure was obviously damaged, which included atrophy and tattered structure of folds, and the intestinal villi were sparse.

In the hindgut, the relative expression of *IL-8* mRNA in the R70 group was significantly higher than that in other groups (*P* < 0.05, Table 9), while *Nrf2* mRNA showed opposite results (*P* < 0.05, Table 9). The relative expression of *IL-10* mRNA upregulated with dietary RM increased (*P* < 0.05, Table 9). The relative expression of *Keap1* mRNA in R30 was significantly (*P* < 0.05, Table 9) higher than that in R50, and lowest in R0 and R70.

## 4. Discussion

### 4.1. Effect of Dietary RM and Tannin on the Growth Performance in Grass Carp

A high level of dietary RM has a negative influence on nutrition absorption and growth performance in aquatic species. According to Ngo et al. [29], 30% of dietary canola meal decreased the growth performance and increased the FCR in barramundi (*Lates calcarifer*). Bu et al. [3] showed that when 20% of dietary fishmeal was replaced by RM, the growth performance, feed intake, and feed efficiency decreased in Ussuri catfish. In Nile tilapia, when 2.5% of dietary hydrolyzable tannin was supplemented, the WR decreased, and FCR increased [30]. In the current study, the WGR did not differ significantly in the practical and semipurified groups. While the FR increased, it might be because RM and tannin affected nutrition metabolism. Hydrolyzed tannins have strong polarity, can form insoluble complexes with protein molecules, reduce protein digestion and absorption rate, and affect protein metabolism [31]. In order to gain enough nutrients, grass carp took in more feeding, and high FCR also proved that.

### 4.2. Effect of Dietary RM and Tannin on Hepatic Health in Grass Carp

High levels of RM injure fish health. Ma et al. [32] reported that 40% of dietary RM increased the activities of serum GOT and glutamic pyruvic transaminase (GTP) in grass carp, and the hepatocytes were damaged, including cytoplasmic vacuolation and cellular rupture [32]. In Ussuri catfish, 37.05% of dietary RM increased serum MDA content and decreased the activities of lysozyme and other antioxidative enzymes [3]. In this study, in hepatopancreas, the MDA content and the activities of some antioxidant enzymes increased with RM level, while the GSH content and GPx activity decreased, indicating that 50% of dietary RM injured the hepatopancreas health and antioxidative capacity of grass carp. A high content of GOP in serum also proved it. GOP content increased if hepatopancreas was damaged [33]. Body health is closely associated with antioxidative capacity, which plays an essential role in removing harmful free oxygen radicals from cells. The high antioxidant potential of tannins has been described in numerous studies [34, 35]. As secondary plant metabolites, tannins are involved in the complex system of antioxidant defense [31]. These strong antioxidant properties are connected with tannins have aromatic rings structure that can be associated with free radical reactions. [36]. Sergediene et al. [37] showed that the antioxidant polyphenols could serve as oxidants. In aquatic species, tannins are toxic and have a prooxidation effect [38, 39]. Rice-Evans et al. [36] observed that 2.5-20 g/kg of dietary tannin increased the contents of serum albumin and globulin in Indian major carp (*Labeo rohita*). These were consistent with *in vitro* experiments when 0.1-11.8 nmol/ml of tannin was added to carp liver homogenate [14]. Similar results were also obtained in the current study. In hepatopancreas, the MDA content and the activities of antioxidant enzymes increased with tannin levels. In serum, the SOD activity increased in the T1 and T2 groups and decreased in the T3 group, while the content of GOT and GPT in the T3 group was higher than those in the T0 group, indicating that 0.75% hydrolyzable tannin impaired hepatopancreas health. GSH has antioxidant properties, and GPx is an important cellular antioxidant enzyme that can reduce oxidative stress caused by reactive oxygen species (ROS) [40]. In the hepatopancreas, the GSH content and GPx activity decreased with dietary tannin levels, which also approved the negative influence on antioxidative ability in grass carp.

Kirby demonstrated that the tannin's prooxidant effects might be attributed in part to the creation of prooxidant intermediates during biotransformation, which induces liver lesions [41]. Gallic acid (GA) may be responsible for it. It is a hydrolysate of hydrolyzable tannin, which can be rapidly nonenzymatically oxidized and generate a great amount of hydrogen peroxide (H_2_O_2_) *in vivo* and induce oxidative stress [42]. The role of SOD is to transform superoxide anion into H_2_O_2_, and GPx and CAT can convert H_2_O_2_ into water [43]. The increasing activity of these three enzymes indicated that more H_2_O_2_ was generated. Tannin can cause hepatic necrosis in mice and grazing animals, indicating that it is a hepatotoxic agent. A significant breakdown of polyribosomes in mouse liver and inhibition of the incorporation of amino acids into hepatic protein was obtained after subcutaneous injections of tannin at 700 mg/kg body weight (BW) because tannin could bind to epithelial proteins, cause precipitation and penetrate through the superficial cells, and damage the liver [44]. A similar result was also observed in Merino ewes. When 1.0 g tannin/kg BW was administrated into the abomasum, the liver showed that midzonal or periacinar coagulative necrosis, abomasal, and kidney were significantly damaged [45]. In the present study, the activity of ACP in the head kidney decreased with dietary tannin, which revealed that the immunity of grass carp might be injured, and the increased lysozyme activity might indicate activation of a protective mechanism necessary to reduce the stress caused by tannin.

In this study, the activities of antioxidative enzymes and relative biochemical index showed a similar tendency in practical and semipurified groups, indicating that 50% RM and 0.75% tannin had adverse effects on the antioxidative capacity in grass carp.

### 4.3. Effect of Dietary RM and Tannin on Intestinal Health in Grass Carp

A high level of plant protein sources injures intestinal health. In rainbow trout (*Oncorhynchus mykiss*), the shorter folds and smaller goblet cell populations were obtained in hindgut when dietary fishmeal was completely replaced by RM [46]. When a diet containing 20% of extracted soybean meal was fed to Atlantic salmon, an inflammatory response was observed, and the proinflammatory cytokines, interleukin 17A (*IL-17A*), interleukin-1 beta (*IL-1β*), and interferon-alpha (*IFN-α*) were significantly upregulated [47]. A similar result was obtained in the current study. The MDA content and SOD activity in the intestine increased, while the GSH content and GPx activity decreased, which indicated that the antioxidative ability in the intestine was injured when 50% of RM was used in the diet of grass carp. Furthermore, the relative expression levels of *IL-8* and *IL-10* were upregulated, while the expression of *Nrf2* was downregulated, and the structure of the hindgut, especially in the R50 and R70 groups, was damaged significantly. These showed that grass carp could tolerate 30% of dietary RM, while 50% of RM caused immune to stress and changed the structure of the intestine.

Tannin has been shown to protect intestinal mucosa from oxidative damage and pathogens, reduce peristaltic activity in digestive problems, and prevent diarrhea in rabbits [48–50]. Liu et al. reported that tannin had positive effects on scavenging ROS and reduced intestinal membrane damage in rabbits [48]. The same results were not observed in the present study in which the structure of the hindgut was injured, and MDA content increased with dietary tannin, indicating that dietary tannin caused oxidative damage and injured the antioxidant system in the intestine of grass carp. The *Nrf2* signaling pathway in the intestine, which affects antioxidant enzyme transcription, was studied for regulating antioxidant enzyme activity at the molecular level [51]. *Nrf2* is anchored to the cytoplasm by connecting with *Keap1*, but it translocates into the nucleus and regulates the transcription of antioxidant enzyme genes in oxidative stress. In mice, improved *Nrf2* mRNA levels can upregulate SOD and CAT expression levels [52]. In our study, dietary tannin upregulated the expression levels of *Nrf2* and SOD activities in the intestine, which indicated that the antioxidant ability of grass carp was damaged, and the upregulation of *Keap1* could reduce the *Nrf2* caused by oxidative stress. The intestinal health was injured in varying degrees in three RM-used groups and three tannin-containing groups. The link between impairment of the intestine and oxidative damage led to the structure of the hindgut being injured, especially in T3.

In fish, the immune status was associated with inflammation, which was initiated and regulated by inflammatory cytokines [53]. In mammals, the upregulation of *IL-8* is correlated with inflammatory intestinal diseases, and anti-inflammatory cytokines (e.g., *IL-10*) are thought to counteract the production of proinflammatory cytokines and limit inflammatory response in endothelial cells in order to prevent inflammation [54]. In the present study, the relative expression levels of *IL-8* and *IL-10* in the tannin-supplemented group were higher than those in the T0 group. These revealed that dietary tannin might induce inflammation in the intestine of grass carp. Similar results were observed in ruminants that hydrolyzable tannin was decomposed to lower molecular weight phenolics in the rumen, which can be absorbed in the intestine and cause toxicity [55].

Several studies in herbivorous fish showed that tannins could oxidize to form high levels of semiquinone radicals and quinones, which would remain in the intestine for the reason of negative charge and high molecular weight [56, 57]. These semiquinones and quinones can generate high levels of ROS in the intestine and result in oxidative stress, lipid peroxidation, alterations of cellular functions, DNA damage, and tumor initiation in aquatic species [39].

From the above results, 0.75% dietary tannin and 50% dietary RM (which contains 1.25% total tannin) have a negative influence on intestinal health, including antioxidative capacity, immune stress, and hindgut structure, and the antioxidative capacity in hepatopancreas was also injured. In this study, grass carp tolerated 30% rapeseed meal (which contains 0.75% total tannin) but could not tolerate 0.75% supplemented tannin. In rapeseed meal, both condensed tannin and hydrolyzable tannin exist, and the structure of tannins in RM may be different from the synthetical tannin used in this study. There is a variety of ANFs in rapeseed meal and practical-diet groups, such as lectin, saponin, and phytic acid. The interactions between various ANFs may be different from single ANF, as these interactions lead to a decrease in the toxic effect of the interacting ANFs [9]. The interactions between tannin and lectin have less impact on antinutritional effects on amylase [58]. Simultaneous consumption of tannin and saponin may induce chemical interactions and inhibit the toxins' absorption from the digestive tract in mice [59].

## 5. Conclusion

In summary, this study demonstrated that 50% rapeseed meal or 0.75% hydrolyzable tannin induced oxidative stress and injured antioxidant ability in both hepatopancreas and intestines. Moreover, it damaged the hindgut structure and resulted in intestinal inflammation in grass carp, which might be ascribed to the *Keap1b*/*Nrf2* pathway in the fish intestine. Hydrolyzable tannin in RM is an important reason for it. Furthermore, a more detailed study is needed to expose the effects of mixtures of ANFs in rapeseed meal in aquatic species.

## Figures and Tables

**Table 1:** Composition and nutrient levels of semipurified diets (air dry basis) %.

Ingredients	T0	T1	T2	T3
Casein	24.00	24.00	24.00	24.00
Gelatin	6.00	6.00	6.00	6.00
Wheat middling	35.00	35.00	35.00	35.00
Corn starch	17.50	17.50	17.50	17.50
Soybean oil	1.40	1.45	1.45	1.45
Ca (H_2_PO_4_)_2_·H_2_O	2.00	2.00	2.00	2.00
Choline chloride	1.00	1.00	1.00	1.00
Vitamins premix^2^	0.50	0.50	0.50	0.50
Minerals premix^3^	1.00	1.00	1.00	1.00
Sodium carboxymethylcellulose	11.60	10.8	10.30	9.80
Hydrolysable tannin^1^	0.00	0.75	1.25	1.75

Proximate composition				
Crude protein	31.01	31.01	31.01	31.01
Crude fat	2.35	2.40	2.40	2.40
Crude ash	2.28	2.32	2.38	2.54
Cellulose	12.20	11.41	10.91	10.42
Gross energy/(MJ/kg)	17.63	17.51	17.42	17.34
Tannin	0.00	0.75	1.25	1.75

1.) Hydrolysable tannins were bought from Wuhan Baixing Bio-Technique Co. Ltd.; effective substance content is 99%. 2) Vitamin premix (mg or IU/kg diet): VA, 6000 IU; VD 3, 2000 IU, VC, 400 mg; VE, 50 mg; VK 3, 5 mg; VB 1, 15 mg; VB 2, 15 mg; VB 3, 30 mg; VB 5, 35 mg; VB 6, 6 mg; VB 12, 0.03 mg; biotin, 0.2 mg; folicacid, 3 mg; inositol, 200 mg. 3) Mineral premix (mg/kg diet): I, 0.4 mg; Cu, 4 mg; Zn, 80 mg; Fe, 150 mg; Mn, 20 mg; Mg, 100 mg; Co, 0.1 mg; Se, 0.1 mg.

**Table 2:** Composition and nutrient levels of practical diets (air dry basis) %.

Ingredients	R0	R30	R50	R70
Fish meal	10.00	0.00	0.00	0.00
Soybean meal	40.00	29.00	15.00	0.00
Rapeseed meal	0.00	30.00	50.00	70.00
Wheat middling	26.00	21.00	18.00	15.40
Wheat bran	10.00	10.00	10.00	10.00
Soybean oil	0.20	0.70	0.80	0.90
Ca (H_2_PO_4_)_2_·H_2_O	2.00	2.00	2.00	2.00
Choline chloride	0.20	0.20	0.20	0.20
Vitamins premix ^1^	0.50	0.50	0.50	0.50
Minerals premix^2^	1.00	1.00	1.00	1.00
Sodium carboxymethylcellulose	10.10	5.60	2.50	0.00
Proximate composition				
Crude protein	31.18	30.00	30.63	30.90
Crude fat	3.89	3.69	3.79	3.89
Crude ash	4.22	4.09	4.04	4.24
Cellulose	12.32	10.94	9.73	9.08
Gross energy (MJ/kg)	17.21	16.90	16.80	16.73
Total tannin	0.16	0.87	1.31	1.75

Note: the vitamin and mineral composition in this table is the same as in Table 1.

**Table 3:** The primers for qPCR.

Gene name	Accession	Forward	Reverse
*IL-8*	JN663841	ACTGAAGCAATGAGTCTTAGAGGT	AGGGTGGCAATGATCTCTGT
*IL-10*	HQ388294	TCTGAAAGTGCTCAGTGCAAA	TCGTCATTGGACTCATAAAACC
*Nrf2*	KF733814	AAGGTGATGCCCTGTCATTC	TAGGTGGAACGGAAACATCC
*Keap1a*	KF811013	GCAAGGACTTCCTGTCCAAG	CCCTCCCGCTATGTAGATGA
*β-Actin*	DQ211096	AAGGCCAACAGGGAAAAGAT	CATCACCAGAGTCCATCACG

**Table 4:** Survival and growth performance of grass carp fed semipurified diets (means ± SD, *n* = 4).

	T0	T1	T2	T3
Initial body weight (g)	8.49 ± 0.32	8.58 ± 0.30	8.34 ± 0.44	8.66 ± 0.40
Final body weight (g)	28.11 ± 2.16	27.89 ± 1.27	26.85 ± 0.25	28.35 ± 0.98
Survival rate%	96.25 ± 4.79	95.00 ± 5.78	98.75 ± 2.50	97.50 ± 2.89
Weight gain rate%	230.96 ± 14.90	225.91 ± 25.79	228.01 ± 20.22	227.85 ± 19.84
Feed conversion ratio	1.87 ± 0.11^a^	1.94 ± 0.08^ab^	2.04 ± 0.07^b^	1.98 ± 0.11^ab^
Feed rate%	3.57 ± 0.14^a^	3.66 ± 0.11^ab^	3.84 ± 0.15^b^	3.75 ± 0.11^ab^
Relative intestine weight%	3.47 ± 0.41	3.43 ± 0.47	3.52 ± 0.51	3.39 ± 0.53
Relative hepatopancreas weight%	1.60 ± 0.08	1.63 ± 0.19	1.69 ± 0.04	1.69 ± 0.13
Relative spleen weight%	0.17 ± 0.01^a^	0.15 ± 0.03^a^	0.12 ± 0.02^b^	0.11 ± 0.01^b^

Notes: in the same line, different lower-case letters indicate significant differences (*P* < 0.05).

**Table 5:** Survival and growth performance of grass carp fed practical diets (means ± SD, *n* = 4).

	R0	R30	R50	R70
Initial body weight (g)	8.53 ± 0.69	8.14 ± 0.97	8.78 ± 0.20	7.79 ± 0.29
Final body weight (g)	55.10 ± 1.73^c^	51.22 ± 0.95^b^	53.60 ± 1.40^c^	46.46 ± 1.03^a^
Survival rate %	95.00 ± 5.78	96.25 ± 4.79	93.75 ± 4.7	97.50 ± 5.00
Weight gain rate%	549.16 ± 18.58	535.40 ± 67.23	496.97 ± 18.58	483.56 ± 53.36
Feed conversion ratio	1.21 ± 0.04^a^	1.31 ± 0.03*a*	1.26 ± 0.03^a^	1.51 ± 0.14^b^
Feed rate %	3.17 ± 0.11^a^	3.40 ± 0.09^b^	3.22 ± 0.09^ab^	3.80 ± 0.21^c^
Relative intestine weight %	3.47 ± 0.41	3.43 ± 0.47	3.52 ± 0.51	3.39 ± 0.53
Relative hepatopancreas weight%	1.77 ± 0.10	1.78 ± 0.09	1.68 ± 0.23	1.59 ± 0.16
Relative spleen weight%	0.13 ± 0.01^ab^	0.11 ± 0.00^a^	0.11 ± 0.01^a^	0.14 ± 0.03^b^

Notes: in the same line, different capital letters indicate significant differences (*P* < 0.05).

**Table 6:** Biochemical and immune parameters in different tissues of grass carp fed semipurified diets (means ± SD, *n* = 3).

	T0	T1	T2	T3
Hepatopancreas				
MDA (nmol/mgprot)	19.06 ± 2.14^a^	56.76 ± 1.63^b^	75.69 ± 1.78^c^	50.36 ± 3.49^b^
SOD (U/mgprot)	134.66 ± 2.33*a*	136.40 ± 3.57^a^	154.68 ± 5.76^b^	147.58 ± 5.5^b^
CAT (U/mgprot)	71.78 ± 1.12^a^	73.60 ± 0.50^ab^	73.56 ± 0.79^ab^	75.68 ± 1.85^b^
T-AOC (U/mgprot)	2.16 ± 0.04^c^	2.34 ± 0.15^d^	1.70 ± 0.06^b^	0.67 ± 0.06^a^
GSH (mg/gprot)	13.06 ± 0.08^c^	12.20 ± 0.06^b^	12.22 ± 0.12^b^	11.45 ± 0.15^a^
GP*x* (nmol/min/mgprot)	64.02 ± 1.91^c^	57.36 ± 1.49^b^	59.37 ± 0.79^b^	50.08 ± 1.61^a^
Serum				
SOD (U/mgprot)	65.93 ± 3.75^a^	98.62 ± 2.84^b^	114.50 ± 6.78^c^	63.78 ± 5.95^a^
T-AOC (U/mgprot)	4.40 ± 0.56^c^	3.41 ± 0.62^b^	1.19 ± 0.26^a^	2.71 ± 0.33^b^
GPT (U/L)	16.78 ± 0.41^ab^	15.78 ± 2.60^a^	20.50 ± 0.31^c^	18.46 ± 0.46^bc^
GOT (U/L)	105.32 ± 2.64^c^	96.38 ± 3.48^b^	81.94 ± 3.08^a^	125.07 ± 7.60^d^
Intestine				
MDA (nmol/mgprot)	18.94 ± 2.15^a^	25.30 ± 1.76^b^	32.90 ± 2.57^c^	35.43 ± 1.24^c^
SOD (U/mgprot)	117.93 ± 5.24^a^	171.90 ± 7.49^b^	182.88 ± 7.65^b^	230.91 ± 4.72^c^
GSH (mg/gprot)	18.79 ± 0.16*d*	17.35 ± 0.62^c^	17.29 ± 0.48^b^	15.58 ± 0.40^a^
GP*x*(nmol/min/mgprot)	71.39 ± 2.77^c^	63.13 ± 1.88^b^	62.15 ± 2.05^b^	50.34 ± 2.79^a^
Head kidney				
Lysozyme (*μ*g/mgprot)	0.11 ± 0.01^a^	0.25 ± 0.02^b^	0.26 ± 0.02^b^	0.54 ± 0.05^c^
ACP (U/mgprot)	2.33 ± 0.02^c^	2.03 ± 0.01^b^	2.00 ± 0.04^b^	0.96 ± 0.01^a^

Notes: in the same line, different lower-case letters indicate significant differences (*P* < 0.05).

**Table 7:** Biochemical and immune parameters in different tissues of grass carp fed practical diets (means ± SD, *n* = 3).

	R0	R30	R50	R70
Hepatopancreas				
MDA (nmol/mgprot)	16.46 ± 1.03^a^	14.42 ± 1.09^a^	32.87 ± 4.98^b^	12.21 ± 4.01^a^
SOD (U/mgprot)	132.91 ± 6.31^a^	139.00 ± 0.68^a^	135.30 ± 2.50^a^	182.59 ± 2.58^b^
CAT (U/mgprot)	98.11 ± 1.70^a^	99.54 ± 1.16^a^	106.62 ± 0.89^c^	101.90 ± 0.63^b^
T-AOC (U/mgprot)	1.20 ± 0.18^a^	2.91 ± 0.30^b^	4.53 ± 0.39^c^	1.17 ± 0.09^a^
GSH (mg/gprot)	16.59 ± 0.12^d^	14.88 ± 0.10^c^	13.62 ± 0.33^b^	10.11 ± 0.11^a^
GP*x* (nmol/min/mgprot)	45.11 ± 0.55^c^	60.41 ± 1.38^d^	37.34 ± 1.20^b^	34.49 ± 2.14^a^
Serum				
SOD (U/mgprot)	102.39 ± 4.68^a^	102.79 ± 4.93^a^	110.06 ± 3.14^a^	131.45 ± 5.05^b^
T-AOC (U/mgprot)	10.32 ± 0.40^d^	9.33 ± 0.51^c^	7.73 ± 0.50^b^	3.66 ± 0.47^a^
GPT (U/L)	18.46 ± 1.62^b^	17.67 ± 0.96^b^	17.72 ± 0.532^b^	15.43 ± 2.40^a^
GOT (U/L)	98.48 ± 2.17^a^	109.39 ± 2.79^b^	103.56 ± 1.14^ab^	127.92 ± 5.83^c^
Intestine				
MDA (nmol/mgprot)	13.03 ± 3.51^a^	23.60 ± 2.72^b^	28.52 ± 1.93^b^	45.04 ± 2.87^c^
SOD (U/mgprot)	171.30 ± 8.06^a^	179.00 ± 7.95^a^	218.16 ± 5.86^b^	263.24 ± 5.91^c^
GSH (mg/gprot)	25.98 ± 0.47^d^	20.55 ± 0.40^b^	21.51 ± 0.63^c^	18.63 ± 0.35^a^
GP*x* (nmol/min/mgprot)	71.46 ± 1.85^c^	68.61 ± 1.86^bc^	64.43 ± 2.67^b^	54.63 ± 2.90^a^
Head kidney				
Lysozyme (*μ*g/mgprot)	0.21 ± 0.01^a^	0.21 ± 0.02^a^	0.39 ± 0.04^b^	0.39 ± 0.02^b^
ACP (U/mgprot)	2.73 ± 0.02^d^	2.63 ± 0.03^c^	2.26 ± 0.03^b^	1.87 ± 0.03^a^

Notes: in the same line, different capital letters indicate significant differences (*P* < 0.05).

**Table 8:** Reactions of mRNA gene expressions in hindgut of grass carp fed semipurified diets (means ± SD, *n* = 4).

	T0	T1	T2	T3
*IL-8*	1.08 ± 0.14^a^	1.39 ± 0.14^b^	1.52 ± 0.18^b^	1.41 ± 0.18^b^
*IL-10*	1.02 ± 0.13^a^	1.37 ± 0.14^b^	1.51 ± 0.17^b^	1.99 ± 0.15^c^
*Nrf2*	1.06 ± 0.13^a^	1.41 ± 0.16^b^	1.64 ± 0.24^b^	1.42 ± 0.13^b^
*Keap1*	0.93 ± 0.09^a^	5.11 ± 0.55^c^	5.88 ± 0.78^d^	2.40 ± 0.35^b^

Notes: in the same line, different lower-case letters indicate significant differences (*P* < 0.05).

**Table 9:** Reactions of mRNA gene expressions in hindgut of grass carp fed practical diets (means ± SD, *n* = 4).

	R0	R30	R50	R70
*IL-8*	0.89 ± 0.10^a^	1.16 ± 0.30^a^	1.08 ± 0.06^a^	1.65 ± 0.28^b^
*IL-10*	0.95 ± 0.12^a^	1.14 ± 0.10^a^	1.36 ± 0.21^b^	1.98 ± 0.18^c^
*Nrf2*	0.98 ± 0.14^b^	0.83 ± 0.10^ab^	0.75 ± 0.11^a^	0.69 ± 0.31^a^
*Keap1*	1.07 ± 0.33^a^	3.35 ± 0.26^d^	2.04 ± 0.45^c^	1.51 ± 0.21^b^

Notes: in the same line, different lower-case letters indicate significant differences (*P* < 0.05).

## Data Availability

Data on the growth performance and several biochemical parameters of T0, T1, T2, and T3 groups have been published in Yao et al. [26]. Data on the growth performance and several biochemical parameters of R0 and R50 groups have been published in Yao et al. [25].
